# Alternative Object Use in Adults and Children: Embodied Cognitive Bases of Creativity

**DOI:** 10.3389/fpsyg.2022.893420

**Published:** 2022-09-02

**Authors:** Alla Gubenko, Claude Houssemand

**Affiliations:** Department of Education and Social Work, Institute for Lifelong Learning and Guidance, University of Luxembourg, Esch-sur-Alzette, Luxembourg

**Keywords:** embodied creativity, affordances, creative process, tool use, action strategies, pretend play

## Abstract

Why does one need creativity? On a personal level, improvisation with available resources is needed for online coping with unforeseen environmental stimuli when existing knowledge and apparent action strategies do not work. On a cultural level, the exploitation of existing cultural means and norms for the deliberate production of novel and valuable artifacts is a basis for cultural and technological development and extension of human action possibilities across various domains. It is less clear, however, how creativity develops and how exactly one arrives at generating new action possibilities and producing multiple alternative action strategies using familiar objects. In this theoretical paper, we first consider existing accounts of the creative process in the Alternative Uses Task and then present an alternative interpretation, drawing on sociocultural views and an embodied cognition approach. We explore similarities between the psychological processes underlying the generation of new uses in the Alternative Uses Task and children’s pretend play. We discuss possible cognitive mechanisms and speculate how the generation of new action possibilities for common objects in pretend play can be related to adults’ ability to generate new action strategies associated with object use. Implications for creativity development in humans and embodied artificial agents are discussed.

## Introduction

Alongside intelligence, with which it shares common mechanisms (Haier and Jung, 2008; Benedek et al., 2014), creativity appears to be an indispensable element for individual flexibility and adaptation (Sternberg, 1999; Collins and Koechlin, 2012). Boden (1998) envisioned creativity on two levels—psychological “P-creativity,” which involves the production of novelty taken on an individual scale, and historical “H-creativity,” which is related to the production of ideas and artifacts that are fundamentally novel for the whole culture, on a historical scale.

Sociocultural (Glăveanu, 2010, 2011, 2020) and 4E cognition^1^ (Malinin, 2019) approaches to creativity suggest that, beyond idea generation, the function of creative cognition is to guide action. Although stemming from different philosophical backgrounds, the sociocultural approach (especially cultural-historical activity theory) and 4E cognition share common methodological endeavors. According to both approaches, human cognition is intertwined with the physical environment—objects, materials, and artifacts developed by humankind (Kaptelinin and Nardi, 2006; Glăveanu, 2013; Malafouris, 2014, 2018)^2^. On both accounts, cognition and action are codetermining. Modeling mental processes is thus not sufficient to understand human cognition; instead, it should be studied in relation to human activity and praxis (Bødker, 1997; Noë, 2004). Notably, an extended cognitive system containing tools becomes a key unit of analysis in the sociocultural approach and 4E cognition paradigm (Leont’ev, 1975, 1978; Vygotsky, 1983; Engeström, 1987; Clark, 1998; Glăveanu, 2013; Favela et al., 2021). Tools and manipulable physical devices are seen as an inseparable part of human cognition, extending users’ sensorimotor capabilities. Sociocultural views stress that a deliberate production of novel and valuable artifacts also relies on shared cultural means and tools, thus extending a personal adaptability account of creativity and assigning it a cultural role of transforming the sociocultural practices (Engeström, 1995, 2001; Miettinen, 1999; Miettinen and Virkkunen, 2005; Glăveanu, 2011).

The view that creativity is intimately related to tool^3^ use is inherent in the Alternative Uses Task (AUT), which psychologists use as a proxy to evaluate human creativity (Wilhelm and Kyllonen, 2021). Yet, the interpretation of important cognitive processes underlying the creative performance in this task has evolved mainly around associative and divergent-convergent accounts. These explanations might be viewed as “disembodied” since they disregard how ideas could be translated into actions and vice versa. Sensorimotor processes associated with object use and the contribution of sociocultural knowledge and conventions to the successful performance in the AUT have been underappreciated in creativity research. Inspired by the ideas of 4E approaches to cognition and sociocultural perspectives, we sketch an alternative, action-oriented and affordance-based interpretation of the performance in the Alternative Uses Task and speculate how sensorimotor embodiment and linguistic and social knowledge may contribute to the development of creative cognition.

In this article, we first review existing interpretations and possible accounts of successful performance in the AUT. We then attempt to interpret the creative process in the AUT through the lenses of embodied cognition and sociocultural approaches. We discuss the role of children’s pretend play in the development of creativity. Our aim is to consider how the interplay between language and action first manifests itself in children’s pretend play and is related to the mental generation of novel and useful action possibilities for common objects in adults. We also discuss implications for creativity promotion in humans and its emulation in embodied artificial agents. We conclude by pointing to the limitations of the work and outlining the future research agenda.

## Existing Interpretations of the Alternative Uses Task

The idea of testing subjects’ creativity by their ability to adapt to situational constraints through improvising solutions and finding a substitute for the missing equipment has been investigated by psychologists since the beginning of the 20th century. Spearman, in his work “Creative mind” (1931), described Strasheim’s (1926) experiments, where children were placed in several situations and had to attain the same goal with different available materials. Guilford and colleagues (Wilson et al., 1953) developed the Alternative Uses Task, which has become a prototypical measure of creativity. In a sense, the AUT could be seen as a reversed Strasheim’s test: instead of fulfilling the same goal with varying objects, the AUT asks subjects to come up with multiple new purposes for the same common object or tool (within a time constraint). The novelty of Guilford’s approach consisted in the claim that what is important in this task is the divergent production—the ability to come up with many diverse, novel, and uncommon responses to an open-ended stimulus. Three metrics have been proposed to score the AUT responses, decomposing creativity into fluency (number of uses), originality (number of unique uses), and flexibility (number of conceptual categories that the generated uses could be binned into; Guilford, 1967; Kaufman et al., 2008).

Despite the “divergent thinking” origin of the AUT, it has been pointed out that the performance on this task also calls for convergent thinking (Wallach and Kogan, 1965; Wallach, 1970; Mumford et al., 1991). Specifically, in addition to idea generation, one has also to select solutions that meet the task’s constraints. Campbell’s early conceptualization of creativity described it as a process of Blind Variation and Selective Retention, which requires “a mechanism for introducing variation, a consistent selection process, and a mechanism for preserving and reproducing the selected variations” (Campbell, 1960, p. 381). According to the Blind Variation and Selective Retention (BVSR) model, the generation of novel uses in the AUT would consist of two distinct processes: an initial generation of uses and subsequent selection based on a person’s internal criteria. Johnson-Laird (1987) described three possible configurations of the BVSR account, which he called Neo-Darwinian, Neo-Lamarckian, and Multi-Stage algorithms^4^. The formalization of Johnson-Laird underscores the role of evaluation in the process of production of creative outcomes and points to the importance of existing domain standards and conventions as potential sources of evaluative metrics.

Another influential explanation of creativity—associative—claims that the generation of new and unconventional solutions stems from the personal ability to fluently *retrieve* and bridge remote concepts and ideas (Mednick, 1968; also Spearman, 1931; Koestler, 1964). According to this approach, the creative ability could be explained by the organization of an individual’s associative networks (Mednick, 1962). More specifically, in low creative people, a given verbal and/or visual stimulus (e.g., pencil) should evoke only salient and conventional items (a pattern Mednick called a *steep associative hierarchy*), whereas, in high creative people, a given verbal input should evoke not just a few salient associations, but also several distantly related ones (a pattern called a *flat associative hierarchy*). These links to more remotely connected concepts would lead to creative and unique answers. Although the theory could elegantly explain associative fluency and originality of solutions in the AUT, the approach remains relatively vague about the origins of such associative and semantic hierarchies (Kenett et al., 2014).

A possible mechanism for identifying correspondences between semantically distant domains was further specified by Gentner (1983). She proposed the concept of analogical *mapping*, which refers to the transfer of similar structural relations^5^ between elements from a known domain or situation to a new one. According to Gentner, a key element in knowledge mapping is similarity comparison between structures of the source and target situations. While Structure Mapping theory explains how analogies are made from already existing structures, the theory still does not answer the question of where the structures come from. The case-based reasoning^6^ approach partially remedies this gap and states that new problems draw on past experience, which is stored as cases and is retrieved, adapted, evaluated, and retained (Kolodner, 1992).

Recognizing the critical role of mapping, Sternberg (1986a,b) and Chalmers et al. (1992) drew attention to the importance of the initial *encoding* of information. They believed that perception must be accounted for in a model of analogical thought and creative cognition. For Davidson and Sternberg (1986), seeing visual stimulus’ features that previously have been nonobvious is crucial for updating the representation of the problem. Applying the analogy account to the solution generation process in the AUT, one of the possible strategies to come up with new uses for a brick would be to notice and encode a relevant feature in a given object (e.g., weight), retrieve knowledge from past experience where a heavy object was used, extract the goal or final effect of this use, and make a mapping of it from known to the current problem.

Benedek et al. (2014) further studied the role of attentional mechanisms that underlie the performance in the AUT. They found that two executive processes—inhibition and updating of the information in working memory—are important for the creative process. It has been argued that while updating helps to keep track of generated ideas and to refresh relevant object features (thus, facilitating idea generation), inhibition helps to filter most common and non-original responses (thus, contributing to idea evaluation and selection).

Finally, a different and insightful account of the performance in the AUT has been proposed by Gilhooly et al. (2007), who redescribed the AUT as a heuristic task that could be accomplished by different cognitive processes. The authors analyzed verbal reports and showed that the task could be solved *via* various heuristic tools—strategies—discovered by participants during the AUT response generation. Notably, these strategies determine the degree of novelty of produced ideas. Gilhooly and colleagues found that whereas an initial generation of uses relies on extant knowledge and its retrieval from episodic long-term memory, the generation of genuinely novel uses is related to more exploratory strategies that occur later in the task.^7^ These later strategies to create novelty are mental decomposition of the object and its property use. The authors suggested that the creation of highly novel uses may rely either on amodal semantic representations or mental imagery—much like in mental rotation and scanning tasks (Shepard and Metzler, 1971; Kosslyn, 1980; Finke, 1989; LeBoutillier and Marks, 2003).

Taken together, the alternative uses in the AUT could be the result of (1) a variety of personal experiences; (2) the ability to retrieve memories; (3) flexible attentional control—the ability to switch attention from one object’s feature to another and inhibit salient responses; (4) mapping abilities; and (5) the ability to discover and make use of strategies facilitating task completion. While the first two factors are related to long-term memory, factors 3–5 are determined by one’s working memory and executive processes (Gilhooly et al., 2007; Nusbaum and Silvia, 2011; Lee and Therriault, 2013; Benedek et al., 2014). A visual summary of the discussed accounts of the cognitive processes underlying the AUT is presented in Figure 1.

**Figure 1:** Cognitive processes posited by existing accounts of creativity. Images sourced with permission from Nfsphoto/Dreamstime; Mishoo/Dreamstime. Other images author’s own creation.

This brief overview highlights that the task demanding the production of creative responses (i.e., combining high novelty and high utility) necessitates recruiting different cognitive processes and developing strategies to achieve the proposed goal. Several important questions remained unanswered, however. Specifically, what is the role of objects in the creative process? How does one come up with novel uses without a real object and the possibility to manipulate it? Ultimately, what exactly do we test with the Alternative Uses Task?

## The Complexity of the AUT and Human Object Use

What makes the Alternative Use Task difficult? First of all, in the AUT agents have to think about an object which is not currently present in the environment. Within the embodied cognition research paradigm this type of task has been called a representation-hungry problem (Clark and Toribio, 1994). The distinction has been made between offline cognition, which takes place in the mental domain and is environmentally decoupled (Wilson, 2002), and online, or situated cognition, which allows manipulating the environment in order to offload cognitive demands into the surroundings.

In the ecological-enactive cognition approach, objects use has been associated with detecting and exploiting information about affordances—what an organism can do with an object or a surface based on its capabilities (Vicente and Rasmussen, 1990; Lockman, 2000; Lockman and Kahrs, 2017; Mangalam et al., 2022). The term “affordance” was coined by Gibson (1979) to emphasize that the agent does not simply process physical properties of the environment (like color, shape, or distance) but rather picks up those dispositional properties that allow to organize the behavior and act on the environment (Rochat and Reed, 1987).

The Torrance Thinking Creatively in Action and Movement test (Torrance, 1981) could be a good example to illustrate the workings of online creative cognition in a concrete situation. The test was conceived by Torrance to rule out the requirement to answer verbally for children starting from 3 years old. As its name suggests, the test allows children to express their creativity through action and body movements with the object. One of the tasks may be seen as an embodied Alternative Uses Task: it invites a child to play with a paper cup and find different uses for it. In contrast to the AUT, which suggests that subjects will create new and original uses for a common object relying solely on unaided mental capacity, the Torrance Test for children provides a real object and allows a direct exploration of its affordances through motor activity. Children can come up with different ways to use the cup by manipulating and examining possible actions and physical properties of the cup (see also Hoicka et al., 2013). Although the test has traditionally been interpreted in terms of fluency and originality of the proposed uses, we suggest that it could alternatively reflect a child’s ability to discover new strategies for action and thus characterize a child’s engagement with a *field of relevant affordances*. Rietveld and Kiverstein (2014) argued that an individual field of relevant *affordances* may be measured by the *scope* of affordances the individual is open to, *salience* of available opportunities for action, and *depth* of anticipation, i.e., how far one can transcend the here and now in planning possible actions (see Rietveld and Kiverstein, 2014; Rietveld et al., 2018 for the description).

Although almost any object offers multiple possibilities for action and potential use, within the members of a given culture object’s use is typically based on a shared convention about the object’s canonical function (Moro and Rodrígez, 2005; Costall, 2012; Dimitrova et al., 2015). This view is inherent in sociocultural theory which claims that beyond providing numerous possibilities for action, human tools and artifacts also represent a way of transmission of cultural knowledge (Leont’ev, 1978; Alessandroni and Rodríguez, 2019). Specifically, according to a sociocultural perspective, the use of human artifacts and tools implies two aspects. The technical aspect refers to the operational side of tool use, with a focus on the properties of the object, its perceptual-motor requirements, and conditions for its implementation in action. The cultural dimension concerns the socially expected or canonical way of performing an action with an object^8^ (El’konin, 1978; Ramstead et al., 2016; see also McNeill, 1992; Clark, 1996; Goldin-Meadow, 2003; Kendon, 2004).

This distinction between an object’s cultural function and other possible operations afforded by the object (Tomasello, 1999) allows capturing the second main source of difficulty in the AUT. The so-called phenomenon of “functional fixedness” entails that the canonical function associated with an object—what the object is commonly used for—may constrain other potential and non-canonical uses and make it difficult to conceptualize and apply the object in other than conventional ways (Luchins, 1942; Duncker, 1945). The phenomenon is thus related to the salience dimension of an individual’s affordances field and manifests itself in the perceptual dominance of a particular action possibility in relation to other affordances.

It has been proposed that an object’s conventional function is inherent in the key meaning of the word used to name it (Awaad et al., 2015; Srinivasan and Rabagliati, 2021). Specifically, Awaad et al.’s (2015, p. 423) insight consists in the proposition that canonical affordances are captured and could thus be “mined” from language dictionaries as “they provide concise and unambiguous definitions of objects that almost always include their function.” This view is in dialogue with a pragmatic perspective, which considers objects’ cultural functions as a conceptual foundation (Alessandroni and Rodríguez, 2019; Alessandroni, 2021). This entails that when reasoning about objects, one typically refers to their conventional affordances. The difficulty to reinterpret the object use may thus be partially explained by language and how one extracts affordances from words.

Experiments in psycholinguistics provide indirect evidence to support this claim. Studies show that the way one categorizes and names the experimental objects affects the principal way how these objects are used (Katz and Hussey, 2011), and what action possibilities are perceived as available. For example, categorizing and naming an object as a “box of tacks” in Dunker’s candle problem (Duncker, 1945) would not allow the same action possibilities as labeling it as a “box and the tacks” and would result in different solution rates.

This hypothesis linking language, affordances, and the conventional way of performing an action suggests that these variables may be equally important and relevant for understanding the performance in the AUT.

**Figure 2:**
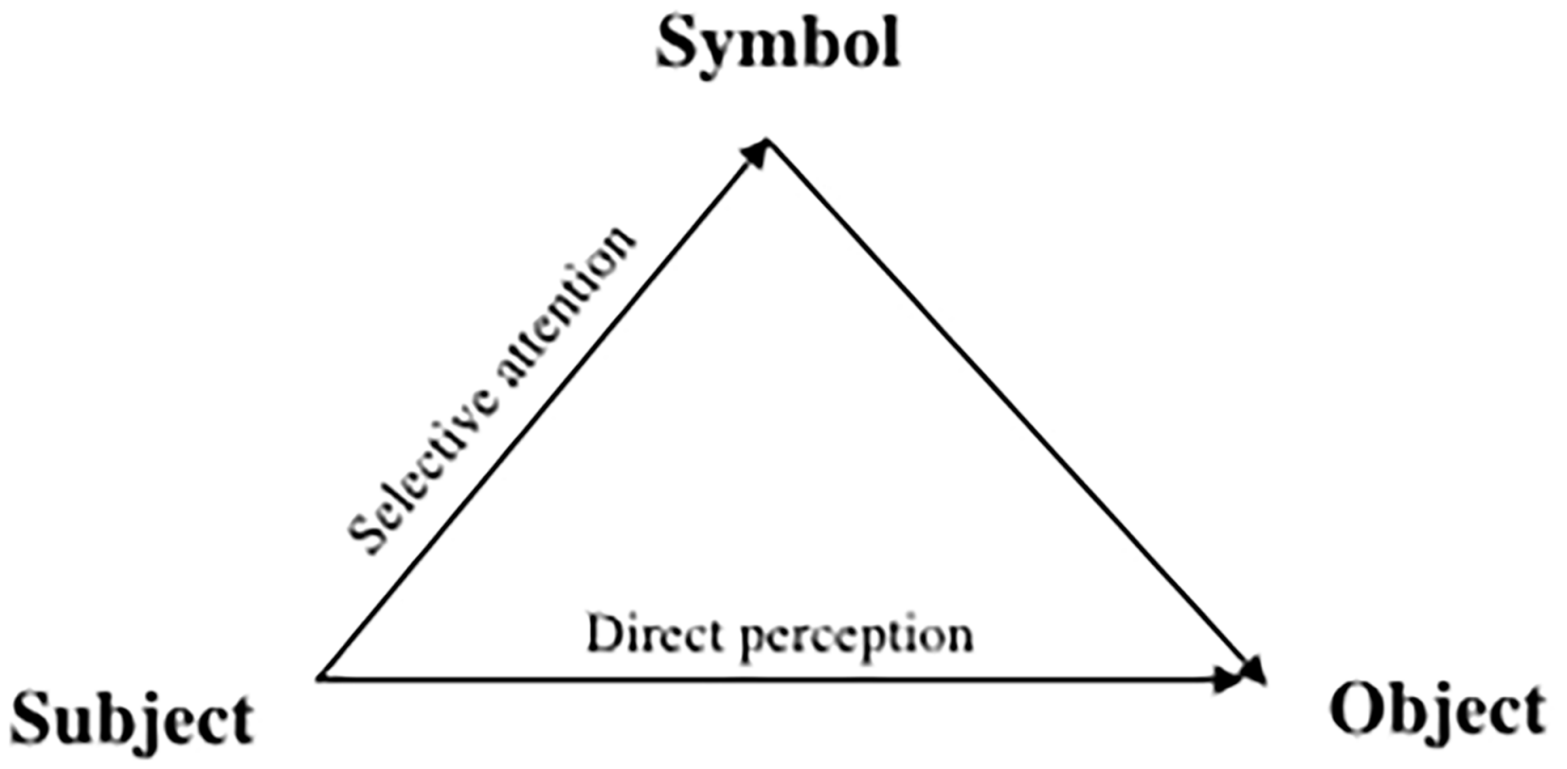
Mediational triangle. Adapted from Vygotsky (1983).

Vygotsky (1983) has expressed the linguistic and cultural mediation of higher mental functions by a scheme (Figure 2) which illustrates how symbols are grounded in perception and suggests that human cognitive processes are vicariant, i.e., functionally substitutable (Cole and Engestrom, 1993; see also Hammond, 1981). In other words, any pursued goal involving object use could be attained by relying on direct perception (Vygotsky called it the “natural route”), on the one hand, and by leveraging crystallized knowledge (the “cultural route,” which includes mediation by sign and symbols), on the other hand.^9^ This schema indicating the existence of dual routes for action (see also Humphreys, 2001) may help to envision a way to bypass the pitfalls and complexities of the AUT.

## The Alternative Interpretations of the AUT

**Figure 3:**
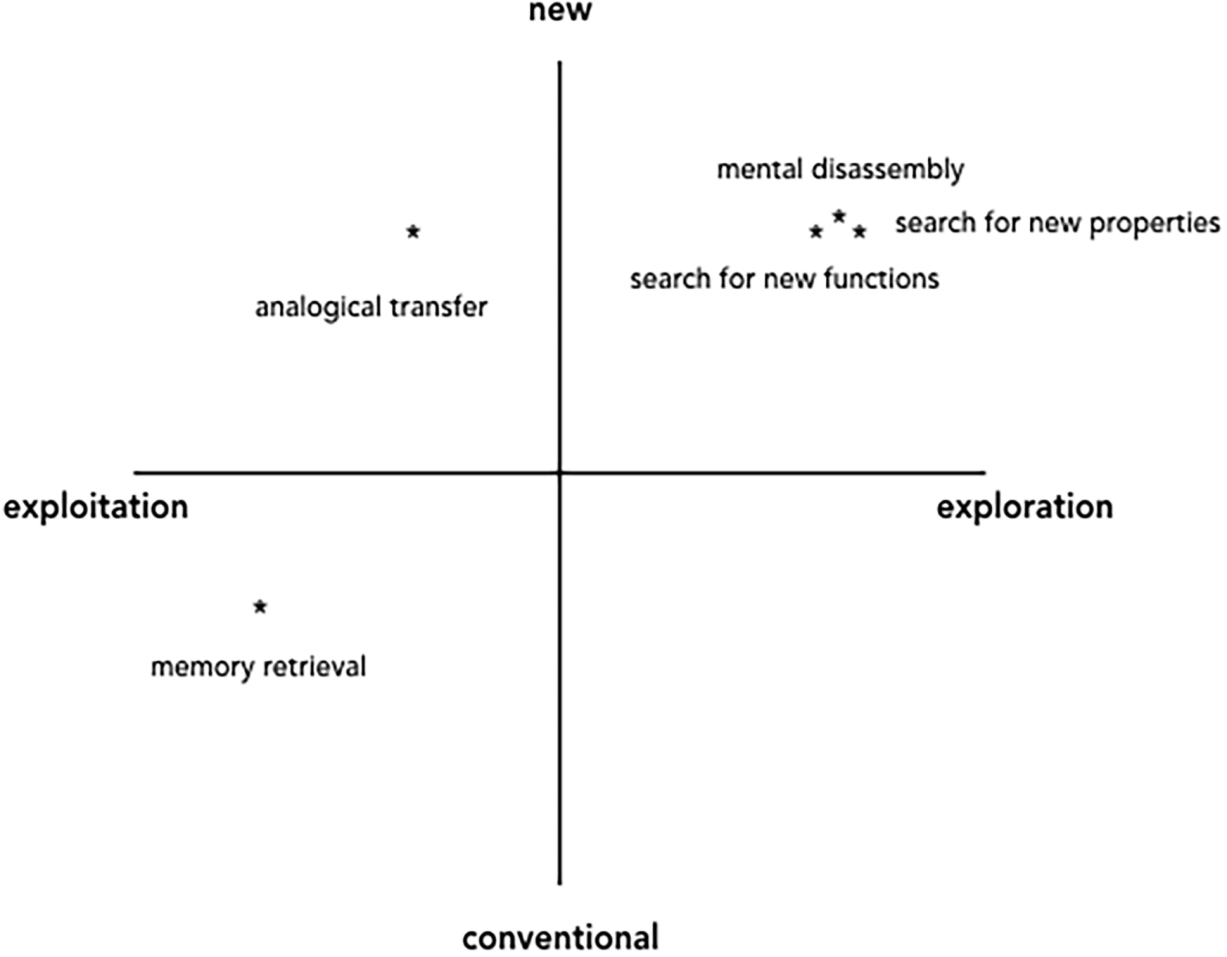
Cognitive strategies used by participants in the Alternative Uses Task (AUT).

Sociocultural, embodied, and enactive accounts of difficulties in the AUT allow us to reconsider the cognitive bases of the AUT. Instead of opting for purely cognitive accounts or suggesting that the Alternative Uses Task tests the ability to come up with new and original ideas, we argue that the AUT actually measures an individual’s ability to create and extend one’s action possibilities with an object. In our view, the process of generating new uses for a common object unfolds as an alternation between *exploitation* of existing knowledge and *exploration of potentialities*. This hypothesis is supported by the findings of Gilhooly et al. (2007), which show that cognitive strategies leading to novel solutions are inextricably action-oriented. They can be viewed as virtual actions, where participants divide and reassemble an object, investigate its properties, and examine potential movements it affords. Figure 3 depicts a possible mapping of strategies on the exploitation-exploration continuum according to the potential novelty of their outcome.

What mechanisms may support the ability to plan and explore potential actions with an object without direct access to it? Three kinds of explanations have been proposed from an embodied cognition perspective (Sanches de Oliveira et al., 2019). According to the first approach, novel uses may consist in the simulation of sensorimotor experience associated with tools (Matheson and Kenett, 2020). Although the simulation used to be considered a potential heuristic to deal with problems involving high uncertainty (Kahneman and Tversky, 1982; Galinsky and Moskowitz, 2000; Ball and Christensen, 2009), Matheson and Kenett (2020) reviewed neuroimaging studies and provided neural evidence of simulations of tool-related actions during the generation of creative uses in the AUT. This data is in line with studies of episodic future thinking (Schacter et al., 2017; see also Schacter et al., 2012), which stress the role of an individual’s ability to recollect past personal experiences. The authors suggested that sensorimotor re-enactment and mental exploration of outcomes of such actions (Matheson and Kenett, 2021; see also Jeannerod, 2001; Witt and Proffitt, 2008) may be an effective strategy to come up with novel alternative uses.

The second approach claims that temporarily scaled-up affordances may support long-horizon planning, the ability to go beyond the here and now and anticipate several steps into the future (Kiverstein and Rietveld, 2018; see also Gibson, 1979; Gallagher, 2017). This idea is reflected in the aforementioned depth dimension of an individual’s set of relevant affordances proposed by Rietveld and Kiverstein (2014) which refers to individuals’ temporal horizon of anticipation and the ability to engage in distal actions. The evidence shows that high creativity is indeed associated with the expertise in imagining distal futures (Meyer et al., 2019).

The third explanation proposed by the Material Engagement Theory radically states that material artifacts mediate, enact, and constitute cognitive processes (Malafouris, 2014, 2018). Creative cognition is thus best understood not as thinking about objects but as creative *thinging*, i.e., thinking with, through, and about materials and things. According to this theory, online cognition (i.e., thinking through and with material things) is not conceptually different from offline cognition (thinking about things) but has a “developmental and evolutionary priority” (Malafouris, 2021, p. 108; see also Gill, 2010).

**Figure 4:**
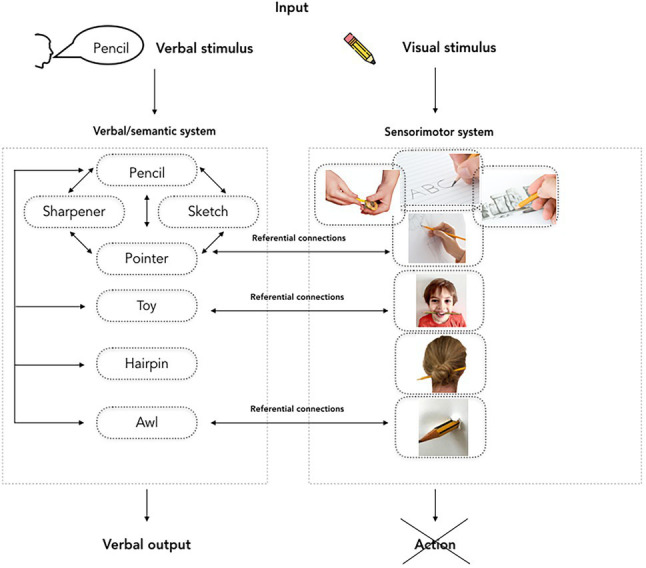
A tentative model of response generation in the AUT. Similar to the naming and action model (Yoon et al., 2002), it posits an interaction between semantic and sensorimotor routes to action. Images sourced with permission from Syda Productions/Adobe Stock; Icefront/Dreamstime; Nfsphoto/Dreamstime; Mishoo/Dreamstime. Other images author’s own creation.

Finally, based on Vygotsky’s idea of two routes of action, we suggest the fourth account, according to which new strategies for action may be the result of combining sensorimotor information related to affordances with the information pertaining to the canonical object use. In other words, language may be used as a tool to reinterpret the meaning of an object and construct new *ad hoc* categories (Barsalou, 1983, 1991; Borghi et al., 2019). A tentative model of response generation in the AUT is shown in Figure 4.

## Pretend Play and the Creation of New Affordances

Vygotsky (1967) argued that children’s play combines symbolic, contextual, and sensorimotor components and may thus hold the key to understanding an imaginative construction of hypothetical events and actions. Below we will follow Vygotsky’s proposal and investigate how the internal mechanics of play could enact creative cognition and contribute to the development of the ability to generate non-conventional uses.

It has been proposed that early prelinguistic sensorimotor explorations of objects’ properties and experience of action on objects are necessary prerequisites for play and creative endeavors (Kravtsova, 1996; Pellegrini and Hou, 2011; Pellegrini, 2013; Nonaka and Goldfield, 2018). This manipulative motor experience allows a child to learn about the effects of their actions on objects (Keen, 2011; Schulz, 2015; Stahl and Feigenson, 2015) and explore the relationship between multiple hidden objects’ affordances, which will be later used in play and creative problem-solving endeavors involving tool use (Smith and Dutton, 1979; Lockman, 2000; Lobo and Galloway, 2008; Adolph and Robinson, 2015; Lockman and Kahrs, 2017). Such active interaction with objects may be seen as the vehicle by which children learn complex action-environment links and means-ends relationships. This idea has been empirically confirmed by Bruner (1975), who showed that the possibility to manipulate materials allowed children to use these materials as tools to solve an unseen practical problem. Bruner observed that playful exploration of materials prior to problem presentation led to problem-solving rates comparable to children who saw how adults performed the task (see also Dansky and Silverman, 1973, 1975; Li, 1978; Dansky, 1980). A similar positive effect of object exploration on test performance has been obtained in the domain of mental rotation. When allowed to perform manual rotations during the training phase, school-aged children performed better at the subsequent mental rotation test (Wiedenbauer and Jansen-Osmann, 2008). Such results suggest that active interaction with objects enacts creative cognition and allows children to build associations between actions and effects in multi-modal sensorimotor spaces and thus better predict the outcome of their actions.

In parallel with self-directed multimodal explorations, children’s direct perception is also shaped by adults. The core mechanism of learning cultural norms and conventional affordances is the synergy of language (Vygotsky, 1983) and joint action (Tomasello, 1999, 2014; Ramstead et al., 2016; Nonaka and Stoffregen, 2020). This synergy allows adults to guide and “educate” children’s attention toward the relevant object’s affordances and demonstrate their socially expected use. As a result, children create referential connections between an object’s conventional function and its name (Nelson, 1974), and by the age of two toddlers become aware that each object has its function (Russ and Christian, 2011, but see Rossmanith et al., 2014 for the evidence that this happens much earlier), recognize and spontaneously produce key (iconic) movements associated with objects’ use (Harris et al., 1993; Rakoczy and Tomasello, 2006; Tolar et al., 2008; Behne et al., 2014), and generalize the objects’ names in accordance with their functions^10^ (Kemler Nelson et al., 2000). Presumably, conventional affordances and socially accepted properties of objects guide an individual’s attention in everyday situations where objects become perceived by their most common action possibilities (Defeyter and German, 2003; Ye et al., 2009).

According to the sociocultural theory, in pretend play two routes of development converge and allow a child to create novelty by leveraging mechanisms of sociocultural conventions and direct perception (Figure 2). The basic feature of play, according to Vygotsky, is the creation of an imaginary situation—a situation where visual and semantic fields diverge (Vygotsky, 1967; Bodrova and Leong, 2007). This feature may be found in one form of pretend play, object substitution, which requires temporarily suppressing the canonical action of the object while performing an action that is typical for another object (Tomasello et al., 1999). When a child enacts an imaginary situation and claims: “This is not a shoehorn, this is a bow!” she demonstrates not only her knowledge of a canonical affordance of the object but also the ability to deliberately transfer it from one object to another (Davydov, 1992; Kravtsova, 1996; see also Rodríguez et al., 2014; Palacios and Rodríguez, 2015). In this process, language plays an important role in allowing the child to manipulate information about an object that is not present in the immediate environment and label her interpretation of the object over the visual field. At the same time, perceptual experience also has an important role in object substitution in pretend play. The mechanism for transfer function from one object to another is not arbitrary. It has been shown that to be used instead of another one, a prop in pretend play should afford the same action or gesture as a missing object^11^ (El’konin, 1978; Vygotsky, 1983; see also Landau et al., 1988; Smith, 2005). Hence, in pretend play involving object substitution, a child has to first perceive a particular affordance of the prop (Dent-Read, 1997; Szokolszky and Read, 2021).

The substitution mechanism could be described in terms of the double-stimulation method^12^ (Vygotsky, 1983; van der Veer and Valsiner, 1991; Engeström, 2007; Sannino, 2015), which implies turning an initially neutral stimulus into a relevant tool. The method has been developed to show that when given a challenging task (first stimulus), children pick up and exploit available materials (secondary or “sign” stimuli) as effective means to solve the task. In pretend play, when resources are limited and the desired object is not available in the immediate environment, children are likely to pick up and exploit affordances of available props. If some object allows for an action similar to that of a missing one, they attribute a linguistic label of the missing entity to this object. For example, if an object (e.g., pencil) affords piercing, it could be reinterpreted as a *needle* or *awl* (Gibson, 1979, p. 133; see also Kemler Nelson, 1999). As a result, in pretense, a child brings into being a new use for a common object based on intended (by the child) and afforded (by the object) action. This mechanism bears resemblance with the aforementioned strategy to generate new uses in the AUT, where a person could pick up an affordance of a common object and then label it with the word corresponding to another object’s canonical affordance.

What competencies related to creativity may the mechanics of pretend play possibly affect? First, in pretend play, children learn that beyond its conventional function and name, a physical object could be designated by different words depending on the intended use and afforded by an object’s action. Language use and associated motor and sensory experiences that arise in multimodal object-oriented play may affect how easily one extracts affordances from words and promote the development of lexical polysemy and behavioral flexibility. This hypothesis is in line with recent evidence showing that children can leverage their sensorimotor (especially visuospatial) experience to infer the new meaning of words (Xu et al., 2017; Starr et al., 2021; see also Srinivasan and Rabagliati, 2021). Thus, beyond playing with objects in pretend play, children are also playing with words and creating new meanings, bridging otherwise “remote” concepts. Word-referent mappings and links created between words’ meanings in pretend play may influence how knowledge is structured and retrieved from memory, and presumably may underlie the aforementioned “flatter” associative hierarchies in creative people (Mednick, 1962; Kenett et al., 2014).

Second, by transferring canonical functions from one object to another, children generate new object substitutions and practice a deliberate creation of new action possibilities for objects beyond their usual uses. This feature has been emphasized as crucially important for creativity (Pellegrini, 2013). Withagen and van der Kamp (2018, p. 1), for example, defined creativity as “the discovery and creation of unconventional affordances of objects and materials.” In a similar vein, Glăveanu (2012) associated creativity with the exploration of environmental affordances otherwise unavailable to the person due to their unperceived, uninvented, or unexploited character.

Third, the discovery and use of nonapparent object proprieties and their potential effects in pretend play could enable building better predictive models of the world (Andersen et al., 2021), leading to better action planning and the ability to mentally enact possible future actions. Inevitably, better abilities to predict the effects of their actions and mentally enact possible future actions would enable better coping with unexpected events.

Finally, playing with objects’ conventional functions may help to further acquire the information about expected and, more importantly, socially *unexpected*, i.e., novel or surprising object applications. Osiurak et al. (2009) have found a strong association between the ability to demonstrate the conventional use of objects and the ability to generate the unusual use for them, suggesting that a correct way of performing an action establishes a kind of benchmark for evaluating novel uses (see also Chylińska and Gut, 2020 for a relevant discussion). In fact, the level of surprise could be defined as the difference between the expected (i.e., normative, conventional) use of an object and the actual use performed by the child. Creative actors could leverage this information about “normativity” (Rietveld and Kiverstein, 2014, p. 332) to predict better the potential surprise of their actions. This may manifest in the deliberate selection among multiple potential object uses of those that would violate expectancies and be original.

To summarize, the interplay between the perception of contextual cues and top-down cognitive influences makes pretend play activity an ideal training ground for the development of creativity and associated cognitive and behavioral flexibility (Dietrich, 2004; Zabelina and Robinson, 2010; Collins and Koechlin, 2012; Diamond, 2013) where the creative actors generate new action strategies using both language and sensorimotor elements (reconstructed or recruited directly from perception).

## Implications for Creativity Development

Our theoretical essay reveals the importance of grounding cognitive strategies for creating novelty in action and perception and underlines the role of cultural conventions and norms in the production of novel uses for common objects. We have reviewed the existing accounts of creativity and suggested that creative cognition could not be well understood without considering the inter-related issues of object use, affordances, and sociocultural conventions (see also Buxbaum et al., 1997; Buxbaum, 2016; Osiurak and Badets, 2016 for related discussions).

We have proposed that whereas the creation of new uses for objects in pretend play could be described as a 2-fold process based on the interplay between the direct exploration of an object’s affordances and conventional knowledge, adults could accomplish the same action mentally *via* planning and mental enacting of multisensory experiences associated with an object (Osiurak and Badets, 2016; Matheson and Kenett, 2020). It is plausible that creative people as measured by the AUT may have a larger repertoire of possible actions or means-ends hierarchies of affordances (Cisek, 2007; Wagman et al., 2016), which allow them to better select actions and anticipate or pick up movements that could produce surprise and yield a novel use. A broader range of possible actions and better access to possible action outcomes could be in part dependent on the amount of action-related information acquired during object exploration and pretend play. It is also plausible that creative individuals could better leverage their sensorimotor (especially visuospatial) experience to create new meanings of words (Xu et al., 2017; Starr et al., 2021), which results in their higher behavioral flexibility.

This work has important implications for the development of models of the creative process and creative expertise. In our view, the real-life creative process unfolds as the alternation between offline cognition, where the person mentally generates ideas, hypotheses and plans, and online cognition, where the actor verifies predictions, but also experiments with the environment’s characteristics and allows materials to guide action (Gubenko and Houssemand, in press; Gubenko et al., in press). Whereas offline creative cognition guides action and unfolds in the mental domain as a form of predictive planning based on existing models of the environment, online creative cognition is linked to the properties of the environment and makes use of it, which allows to build better cognitive models of the world and generate better hypotheses (Spiridonov et al., 2019; see also Klahr and Dunbar, 1988). Although the performance in the offline mode is dependent on one’s executive capacities, offloading of cognitive demand *via* external manipulations and tool use in the online mode may alleviate individual differences in working memory (Armitage and Redshaw, 2022). This observation may account for the low association between measures of offline divergent thinking and real-life creative achievement reported in the literature (Zabelina, 2018).

**Figure 5:**
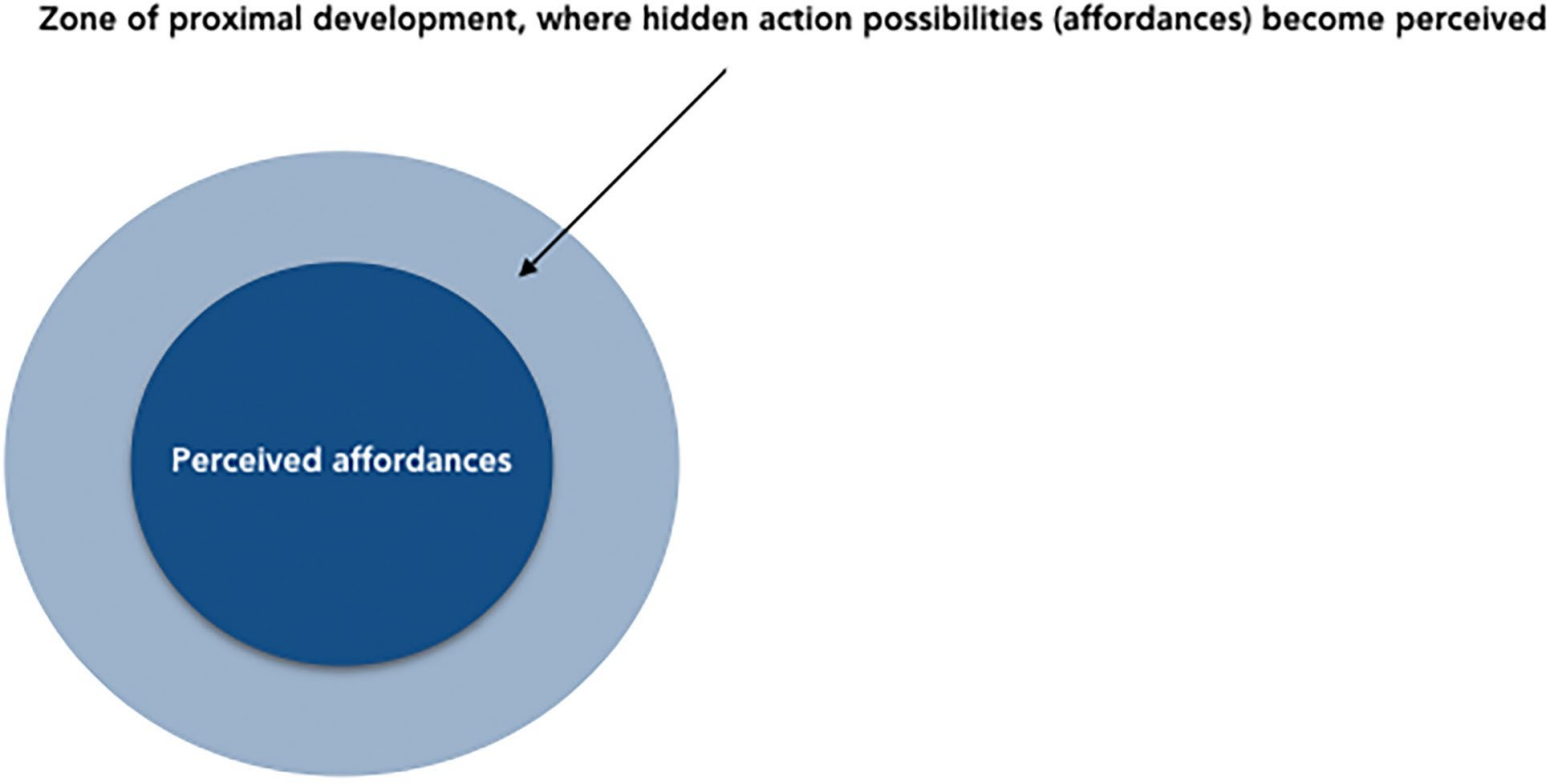
The zone of proximal development as an extension of children’s action possibilities.

Turning to the development of creative expertise, the critical role of material objects, embodied interactions, and crystallized knowledge stresses the necessity to address both “natural” and “cultural” routes in fostering creativity. In addition to interventions based on language-derived (symbolic, amodal) knowledge combination and idea generation, we find it necessary to include object-oriented activities with a high degree of motor-sensory experience and perceptual learning (e.g., construction, educational robotics, hands-on scientific experimentations) into programs aiming at creativity development. Self-directed explorations in conjunction with formative interventions, where instructors would guide and direct learners’ attention to hidden means-ends links and action possibilities, could target the so-called zone of proximal development (Vygotsky, 1978). In such interventions, hidden affordances become learners’ potential action strategies (Figure 5).

The critical role of action on objects for the development of creative cognition suggests that embodied and socially situated autonomous agents may significantly complement symbolic computer systems aiming to emulate creativity computationally (Pezzulo et al., 2013; Lubart et al., 2021). Of particular interest is the research in developmental and cognitive robotics, which underscores the role of embodied interactions and human scaffolding in the development of cognitive competencies in artificial agents (Fong et al., 2002; Lindblom and Ziemke, 2003; Lungarella et al., 2004; Dautenhahn, 2007; Cangelosi et al., 2015; Kumar et al., 2021). Recent works on modeling language grounding (Roy et al., 2004; Krunic et al., 2009; Shridhar et al., 2020), robotic creative (Antunes et al., 2016; Nyga et al., 2018; Nair and Chernova, 2020) and conventional tool use (Awaad et al., 2015; Chu et al., 2016; Neemeh, 2019), as well as the ability to transfer objects’ functions (Agostini et al., 2015) could help to verify and test the models of the creative process and further unveil the role of visual perception, motor components and language in the genesis and development of creativity.

## Conclusion

In this paper, we have attempted to look at the creative performance in the AUT from a different perspective and place the concept of creativity into a broader theoretical context. We have argued that possible interpretations of the creative process should include an effort to reveal the underlying mechanisms and trace their origins. While not being mutually exclusive with existing accounts of creativity, we believe that some insights drawn from the sociocultural perspective and 4E paradigm may extend and augment the existing views of the creative process.

Inevitably, this enterprise required a good deal of speculation. Although presenting a theoretical decomposition of the task, the effort was made toward a functional and, hopefully, more holistic and enriched view of creativity. Synergistic efforts of cognitive and developmental psychologists and scientists in computational and robotic creativity could extend the further research agenda and empirically validate the outlined ideas.

## Data Availability Statement

The original contributions presented in the study are included in the article/supplementary material; further inquiries can be directed to the corresponding author.

## Ethics Statement

Written informed consent was obtained from the individual(s), and minor(s)’ legal guardian/next of kin, for the publication of any potentially identifiable images or data included in this article.

## Author Contributions

AG conceived and wrote the manuscript. CH supervised the work, revised the manuscript, and commented on the text. All authors contributed to the article and approved the submitted version.

## Conflict of Interest

The authors declare that the research was conducted in the absence of any commercial or financial relationships that could be construed as a potential conflict of interest.

## Publisher’s Note

All claims expressed in this article are solely those of the authors and do not necessarily represent those of their affiliated organizations, or those of the publisher, the editors and the reviewers. Any product that may be evaluated in this article, or claim that may be made by its manufacturer, is not guaranteed or endorsed by the publisher.

## Footnotes

^1^The 4E cognition aligns four contemporary approaches in human cognition—embodied, embedded, enactive, and extended—under one heading (Newen et al., 2018).

^2^The relation between cognition and material culture is at the fore of Cultural-Historical Activity Theory (CHAT) and Material Engagement Theory (MET). Using different vocabularies, both theories state that cognition is mediated and constituted by material objects and place it in human activity in which the individual is engaged (Bødker, 1997; Malafouris, 2018). CHAT and MET deny the opposition of mind and matter, psychological and material (Leont’ev, 1975; Malafouris, 2019). A key notion of “thinging” which in MET stands for “the active participatory process by which things are presented to us through acts of engagement and enactive participation” (Malafouris, 2014, p. 143) is very close to the notion of “object-orientedness” in CHAT. In CHAT, human activity is always directed at a certain object, and “the mind emerges, exists and could be understood only in the context of the subject-object relationship” (Leont’ev, 1975; Kaptelinin and Nardi, 2006, p. 66).

^3^Here and further in the text, we use the term “tool” in the sense of an artifact, i.e., a material object with a specific function (e.g., brick, newspaper, and pencil).

^4^Although Johnson–Laird proposed that the Neo-Lamarkian process best corresponds to the creative process, we suggest that all three could coexist and co-occur in a real-life creative endeavor. Likewise, the algorithm could be a function of expertise and evolve over time.

^5^Gick and Holyoak (1983) argued that it could be goals that are transferred, while Carbonell (1983) pointed to the role of relations.

^6^In contrast to cross-domain mappings suggested by Structure Mapping theory, Case-Based reasoning can be considered a form of intra-domain analogy.

^7^This finding suggests that motivation and personality traits, such as perseverance (Sternberg and Lubart, 1995) or openness to experience (McCrae, 1987), play an important part in the creative process, allowing a person to continue the search when resources of experiential strategy have been exhausted.

^8^This canonical way of using an object or tool is usually demonstrated to a child by an adult in early triadic infant-caregiver-object interactions (El’konin, 1978; Rossmanith et al., 2014; Palacios and Rodríguez, 2015; Nonaka and Goldfield, 2018; Belza et al., 2020).

^9^“[T]he emergence of mediated action does mean that the mediated path replaces the natural one … the incorporation of tools into the activity creates a new structural relation in which the cultural (mediated) and natural (unmediated) routes operate synergistically” (Cole, 1996, p. 119).

^10^See also Alessandroni (2021), who argued that canonical uses should be treated as enactive cases of conceptual thinking through which children extend functional behaviors. Along this line of reasoning, functional-based generalizations may occur beyond object names.

^11^See also Ye et al. (2009) for the same pattern in adults’ object substitution.

^12^The method of double stimulation is referred to with different terms such as experimental genetic method, functional method of double stimulation, functional method of twofold stimulation, and historical–genetic method (Sannino, 2015).
